# Intensive Care Admissions for Severe Chikungunya Virus Infection, French Polynesia

**DOI:** 10.3201/eid2404.161536

**Published:** 2018-04

**Authors:** Adrien Koeltz, Stephane Lastere, Sylvain Jean-Baptiste

**Affiliations:** Taaone Hospital, Papeete, French Polynesia (A. Koeltz, S. Lastere);; Bichat Hospital, Paris, France (S. Jean-Baptiste)

**Keywords:** Chikungunya, arboviruses, encephalopathy, Guillain-Barré syndrome, myocarditis, sepsis, intensive care, French Polynesia, viruses, vector-borne infections, CHIKV

## Abstract

During the 2014–2015 chikungunya outbreak in French Polynesia, 64 patients with confirmed chikungunya virus infection were admitted into intensive care. Sixty-three were nonpregnant adults; 11 had an atypical form, 21 had severe sepsis or septic shock, and 18 died. These findings indicate that critical illness frequently complicates the course of chikungunya virus infection.

The first case of chikungunya virus (CHIKV) infection in French Polynesia (Tahiti) was diagnosed in May 2014; it was imported from Guadeloupe Island in the Caribbean (*1*). During the outbreak that developed during October 2014–March 2015, ≈25% of the local population (272,000 residents) became infected with CHIKV (*2*). French Polynesia has 2 potential mosquito vectors for CHIKV: *Aedes aegypti* and *A. polynesiensis.* Phylogenic analysis showed that the French Polynesia strain of CHIKV belongs to the Asian lineage and is closely related to the strain collected in Guadeloupe and the British Virgin Islands in 2014, showing 99.9% homology with that strain (*3*). To describe patient characteristics and clinical courses of chikungunya patients in French Polynesia during 2014–2015, we retrospectively reviewed the medical files of all patients with documented CHIKV infection.

CHIKV infection was defined by the association of compatible symptoms of fever and arthralgia and positive IgM serology or positive blood reverse transcription PCR (RT-PCR). We defined types of CHIKV infection as follows: 1) common form (i.e., only fever or arthralgia); 2) atypical form (i.e., involvement of >1 organ systems); and 3) severe form (i.e., failure of >1 organ systems or admission to an intensive care unit [ICU]).

We used standard definitions for organ system failures and severe sepsis shock (*4*). Organ failures were defined by a Sequential Organ Failure Assessment score >3 for each organ. Encephalitis was defined in accordance with Consensus Statement of the International Encephalitis Consortium criteria (*5*) and myocarditis in accordance with Position Statement of the European Society of Cardiology criteria (*6*).

During the outbreak, CHIKV was confirmed in 63 adults and one 11-year-old girl (Table). Forty-two patients had positive results for blood RT-PCR, and 22 had positive results for IgM serology. Virus load in serum was high; median load was 7.52 log_10_ copies/mL (interquartile range 3.47–9.39 log_10_ copies/mL). Of 5 patients with encephalitis symptoms, 3 had positive results for cerebrospinal fluid RT-PCR.

**Table:** Clinical and laboratory characteristics of 64 patients with chikungunya virus infection admitted into the intensive care department, French Polynesia, 2014–2015*

Characteristic	Result
Baseline	
Median age, y (IQR)	62 (49–71)
Sex, no. (%)	
M	37 (58)
F	27 (42)
Preexisting disease, no. (%)	
Hypertension	37 (58)
Diabetes mellitus	22 (34)
Chronic renal failure	15 (23)
Chronic heart failure	12 (19)
Chronic liver disease	3 (5)
None	15 (23)
Simplified Acute Physiology Score (IQR)	48 (28.5–68.5)
Chikungunya diagnosis, no. (%)	
By reverse transcription PCR	42 (66)
By IgM	26 (41)
By reverse transcription PCR and IgM	4 (6)
Finding at admission	
Organ failure,† no. (%)	
Hemodynamic	40 (63)
Renal	30 (47)
Neurologic	20 (31)
Respiratory	33 (52)
Hepatic	16 (25)
Hematologic	9 (14)
Laboratory‡	
Leukocyte count, cells/m^3^, median (IQR)	11,600 (7,200–15,200)
Lymphocyte count, cells/m^3^, median (IQR)	1,000 (600–1,500)
Lymphopenia, <1,000 cells/m^3^ (% of patients)	36 (56)
Platelet count, cells/m^3^, median (IQR)	155,000 (79–208)
Platelet count, <150,000 cells/m^3^ (% of patients)	34,000 (53)
Creatinine, μmol/L, median (IQR)	132 (79–184)
Creatine phosphokinase, mmol/L, median (IQR)	222 (124–1,160)
Alanine aminotransferase, UI/L, median (IQR)	35 (19–76)
C-reactive protein, mg/L, median (IQR)	10.6 (2.8–18.3)
Procalcitonin, μg/L, median (IQR)	1.72 (0.42–18.3)
Lactate, mmol/L, median (IQR)	2.6 (1.1–5.4)
Chikungunya reverse transcription PCR‡	
Viral load in serum, log_10_ copies/mL (IQR)	7.52 (3.47–9.39)
Viral load in cerebrospinal fluid,§ log_10_ copies/mL (IQR)	4.18 (3.86–4.26)
Outcome variable	
ICU length of stay, d, median (IQR)	3 (2–7)
Crude intensive care unit deaths, no (%)	18 (28)

*IQR, interquartile range. †Organ failure is defined according to a Sequential Organ Failure Assessment score >3 for each organ. ‡Blood samples during the first 24 h. Reference values are as follows: leukocytes, 4.0–10.0 × 10^3^ cells/mm^3^; lymphocytes, 1.5–3.4 × 10^3^ cells/mm^3^; platelets, 150–400 × 10^3^/mm^3^; creatinine, 0.56–1.0 mg/dL; creatine phosphokinase, 0–130 U/L; alanine aminotransferase, 0–35 U/L; C-reactive protein, <5 mg/L; procalcitonin, <0.5 ng/mL; lactate, 5–15 mg/dL. §Virus load was positive for 5 of the 9 cerebrospinal fluid samples.

Forty-nine (76%) patients had a preexisting disease, 33 (51%) required invasive mechanical ventilation, 40 (62%) were in shock and needed vasoactive drugs, and 30 (46%) required renal replacement therapy. The ICU death rate for chikungunya was 28%, slightly higher than the usual 22% ICU death rate (A. Koeltz, unpub. data). Five patients had encephalitis, 2 had myocarditis, and 4 had Guillain-Barré syndrome (GBS). Fifty-five patients had a severe form of chikungunya, and 21 had illness consistent with the case definition for severe sepsis; for 2 patients, no other cause for GBS than CHIKV was identified. Two patients had CHIKV–leptospirosis co-infection, and 1 had CHIKV–dengue virus co-infection. Among the 55 patients who had the severe form of chikungunya, 17 had exacerbations of a chronic condition.

Chikungunya can be complicated by severe multiple organ failure and lead to death either from exacerbation of a preexisting disease or by severe atypical infection. Severe septic shock directly attributable to CHIKV was reported during the 2014 outbreak (*7*,*8*), and these reports seem consistent with our study (2 cases). This finding could be explained by the fact that chikungunya induces lymphopenia.

Neurologic complications of arbovirus infections are well documented, as illustrated by the high incidence of GBS reported during French Polynesia’s outbreak of Zika virus (42 cases) (*9*). In our study, we observed 4 severe cases of GBS, and 10 GBS cases were managed in the hospital during the outbreak; GBS incidence was 4 times higher than usually observed in this hospital.

The most severe atypical complication in our study was myocarditis (2 cases), which had a 100% case-fatality rate. These deaths included an 11-year-old child and a 56-year-old woman without preexisting disease. 

Our findings indicate that critical illness frequently complicates the course of CHIKV infection. Hospitals in chikungunya-endemic areas should be aware of the potential for increases in the number of ICU admissions during outbreaks.
